# A systematic review and meta-analysis of randomized controlled studies comparing follicular flushing versus aspiration during oocyte retrieval in IVF cycles

**DOI:** 10.1186/s40834-025-00351-w

**Published:** 2025-03-31

**Authors:** Nour A El-Goly, Ahmed M Maged, Aimy Essam, Amira Shoab

**Affiliations:** 1 Faculty of medicine, Cairo University, Cairo, Egypt; 2 Department of Obstetrics and Gynecology, Kasr Al-Ainy Hospital, Cairo University, Cairo, Egypt; 3 11 Eid Mostafa Street, Haram, Giza, Egypt

**Keywords:** Follicular flushing, Follicular aspiration, Ovum pick up, Retrieved oocytes, IVF, Clinical pregnancy rate

## Abstract

**Objectives:**

To assess the value of follicular flushing during ovum pick up compared to follicular aspiration in IVF cycles.

**Search strategy:**

Screening of PubMed, Web Of Science, Cochrane, Scopus, and clinical trials registry from inception to October 2024. The search key words included follicular flushing, follicle aspiration, ovum pick up, oocyte retrieval, IVF, and their MeSH terms.

**Selection criteria:**

This review included all RCTs that evaluated the use of follicular flushing during ovum pick-up. Seventeen studies including 2218 participants (1124 were subjected to follicular flushing and 1094 subjected to follicular aspiration) were included.

**Data collection and analysis:**

The extracted data included the settings of the study, the number and characteristics of participants, intervention details including the number of flushes, and the suction pressure used, outcome parameters including number of retrieved oocytes, the oocyte/ follicle ratio, the number of MII oocytes, the time of the procedure, the fertilization, implantation, clinical pregnancy, chemical pregnancy, ongoing pregnancy, live birth, miscarriage and cancellation rates, and risk of bias assessment.

**Main results:**

The number of retrieved and MII oocytes were evaluated in 14 and 11 studies with 1920 and 1588 participants and revealed a mean difference (MD) of 0.03 and 0.16 with [-0.50, 0.57] and [-0.29, 0.61] 95% CI (*P* value =0.9 and 0.48, I^2^ = 87% and 90%), respectively.

The fertilization and implantation rates were evaluated in 4 and 7 studies with 3331 and 1605 participants and revealed an Odd Ratio (OR) of 1.48 and 0.91 with [0.98, 2.24] and [0.55, 1.51] 95% CI (*P* value =0.06 and 0.72, I^2^ = 82% and 61%), respectively.

The clinical pregnancy rate was evaluated in 11 studies with 1542 participants and revealed an Odd Ratio (OR) of 1.23 with [0.86, 1.74] 95% CI (*P* value =0.26, I^2^ = 42%).

The ongoing pregnancy /livebirth rate was evaluated in 11 studies with 1266 participants and revealed an Odd Ratio (OR) of 1.07 with [0.80, 1.43] 95% CI (*P* value =0.65, I^2^ = 0%).

The time of the procedure was evaluated in 8 studies with 985 participants and revealed a mean difference (MD) of 178.58 with [98.23, 258.93] 95% CI (*P* value <0.001, I^2^ = 97%).

The cycle cancellation rate was evaluated in 5 studies with 856 participants and revealed an Odd Ratio (OR) of 0.66 with [0.45, 0.98] 95% CI (*P* value =0.04, I^2^ = 0%).

**Conclusion:**

Follicular flushing during oocyte retrieval did not improve the number of retrieved oocytes, the oocyte retrieved over the aspirated follicles ration, the number of MII oocytes, the fertilization rate, implantation rate, clinical pregnancy, chemical pregnancy, ongoing pregnancy/livebirth, and miscarriage rates and associated with significant prolongation of the procedure. Cycle cancellation was significantly improved with follicular flushing in women with poor ovarian response.

**Trial registration:**

Registration number CRD42024600698 date of registration 23/10/2024.

## Introduction

IVF is a relatively complicated procedure that involves a series of stages. The number of oocytes obtained after the hormonal ovarian stimulation is very crucial in determination of IVF success [1].

Initially, ovum pick up was challenging and performed by either laparotomy or laparoscopy with less than 50 % success rate [2].

This rate was improved with the introduction of foot-controlled suction pressure control [3], and Teflon lined beveled aspiration needles [4].

Ovum pick up is usually performed under general anesthesia after 34 -38 hours of ovulation triggering [5].

The role of first come first serve is usually followed during ovum pick up to avoid intraovarian bleeding, inadvertent follicular rupture, and to ensure continuous visualization of the needle during aspiration to avoid pelvic organs and vessels injury [6].

Although ovum pick up is a relatively safe procedure, it may be associated with pain, infection (0.6%), vaginal bleeding (8.6%), and complications of the used anesthesia [7].

Several modifications were suggested to maximize the number of retrieved oocytes during ovum pick up especially in women with poor ovarian response [8]

The use of follicular flushing was introduced to reduce the risk of oocyte retention. However, the use of flushing may have a damaging effect on the retrieved oocytes. While some investigators suggested the use of follicular flushing in all women, others restricted its use to poor responders and another group rejected its use in all cases.

Older non RCTs suggested that follicular flushing increased the number of retrieved oocytes [9–11].

Subsequent studies yielded conflicting results regarding the benefits and risks of follicular flushing [12].

So, the conduction of this review was necessary to search for evidence regarding follicular flushing use during ovum pick up.

### Objective

To evaluate the safety and efficacy of value of follicular flushing compared to follicular aspiration during ovum pick up in IVF cycles.

## Methods

This study was prospectively registered following the PRISMA guidelines of randomized controlled studies with CRD42024600698 number.

### Eligibility criteria, information sources, search strategy

Two authors independently searched the different databases including PubMed, Web Of Science, Cochrane, Scopus, and clinical trials registry from inception to October 2024. The search key words included follicular flushing, follicle aspiration, ovum pick up, oocyte retrieval, IVF, and their MeSH terms.

### Study selection

This review included all RCTs that evaluated the use of follicular flushing and compared it to follicular aspiration during ovum pick-up step in IVF cycles without language restrictions. It included all studies regardless of the number of flushes, the suction pressure used and, in all participants, whether poor, normal, or high ovarian responders.

After completing the search, the same 2 authors independently screened the articles for possible inclusion in this review. Any disagreement between them was reviewed and evaluated by all other authors.

After establishment of the included studies, 2 authors independently extracted the data from the selected articles using an extraction data sheet. The sheet included the settings of the study, the number of randomized and analyzed participants, the inclusion and exclusion criteria of the participants, all the intervention details including the number of flushes, and the suction pressure used, outcome parameters including both primary and secondary ones, risk of bias assessment and trial registration details.

The reported outcomes included the number of retrieved oocytes, the oocyte/ follicle ratio, the number of MII oocytes, the time of the procedure, the fertilization, implantation, clinical pregnancy, chemical pregnancy, ongoing pregnancy, live birth, miscarriage, and cancellation rates.

The risk of bias assessment for the included studies followed the recommendations of the Cochrane Handbook of Systematic Reviews for evaluation of RCTs. These recommendations included assessment of the random sequence generation, allocation concealment, participants and outcome assessor blinding, incomplete and selective data reporting and assessment of other biases. GRADE analysis was used to assess the quality of evidence for each outcome. GRADE assessment included the number of the reporting studies, risk of bias, inconsistency of the reported outcome, indirectness of data, sample size, width of CI and publication bias.

### Statistical analysis

The overall effect estimate for dichotomous and continuous variables was done through measurement of Odd Ratio and the mean differences with 95% CI for both, respectively. The fixed or random effect models were used in non-significant and significant studies heterogeneity, respectively. The heterogeneity was evaluated through assessed by Cochran’s Q test and I^2^ statistics. The level of significance was set at or below 0.05 for P value and at or above 40% for I^2^. All statistical calculations and subgroup analysis were done using the Review Manager (RevMan) version 5.4.1 (The Nordic Cochrane Centre, Cochrane Collaboration, 2020, Copenhagen, Denmark).

## Results

**Fig 1:**
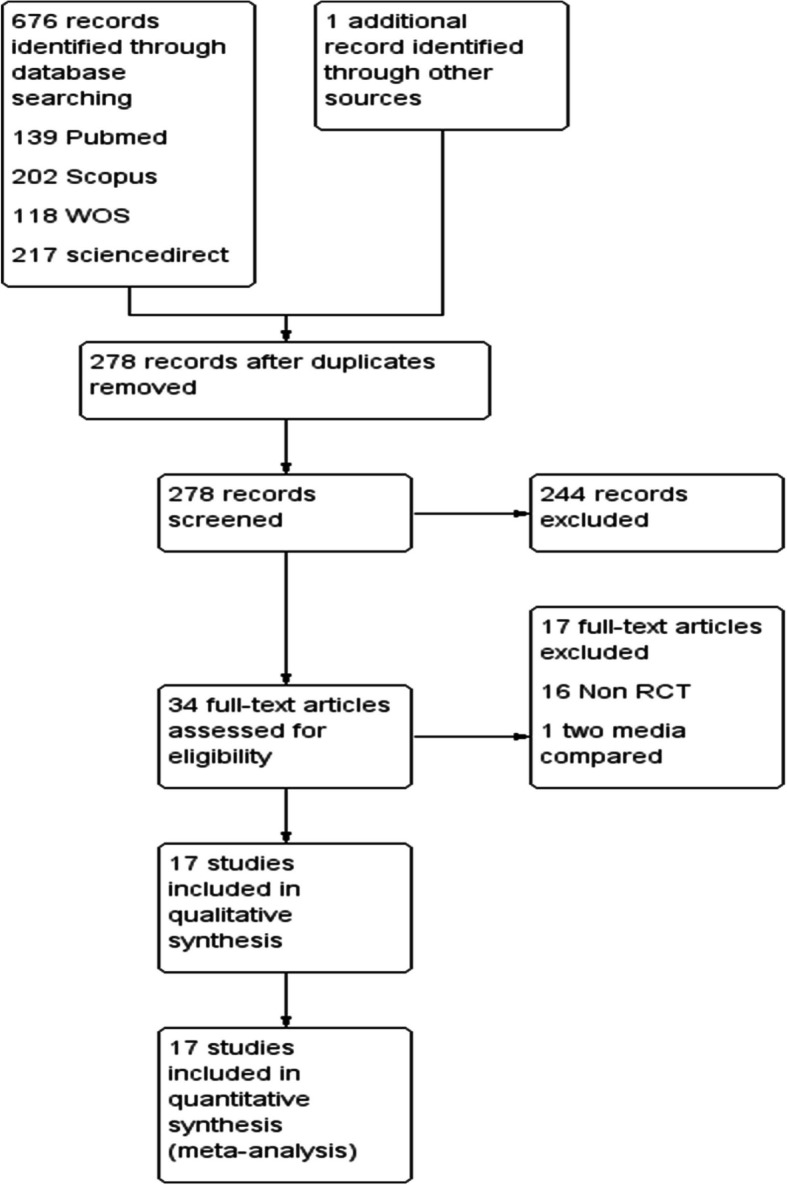
PRISMA flow chart

Study selection, study characteristics:

The flow chart of the search process is shown in Figure 1.

Seventeen studies including 2218 participants (1124 were subjected to follicular flushing and 1094 subjected to follicular aspiration) were included in our meta-analysis [13–29].

All the included studies were published in English language and conducted in a single center.

Four studies were conducted in USA [21–23, 27], 3 in Turkey [16–18], 2 in UK [19, 28] and one study was conducted in each of the following countries Australia [15], Brazil [14], Egypt [25], France [13], Germany [29], Italy [24], Greece [20] and Switzerland [26].

The included participants were poor ovarian responders in 8 studies [13, 14, 17, 21–23, 26, 29], normal responders in 1 study [16] and unspecified in relation to ovarian response in 8 studies [15, 18–20, 24, 25, 27, 28]. The suction pressure used in the included studies ranged between 80 mmHg and 220 mmHg. In 1 study the suction pressure was manually determined [27] and unspecified in 6 studies [15, 16, 18, 19, 23, 24].

The number of follicular flushes were 1 flush in 5 studies [13, 16, 18, 21, 27], 2 flushes in 2 studies [24, 25], 3 flushes in 4 studies [14, 17, 22, 29], 4 flushes in 1 study [23], 5 flushes in 4 studies [15, 19, 20, 26] and 6 flushes in 1 study [28].

The included studies characteristics including the settings, sample size, participants characteristics, details of interventions, study outcomes and trial registration details are presented in Table 1.

**Table 1:** Characteristics of the included studies

Study	Settings	Sample size	Participants	Interventions	Outcomes	Other
Calabre 2020 [13]	Single France	252 randomized 252 analyzed	Inclusion criteria: Age < 43 years married or partnership > 2 years produced < 5 follicles > 14 mm on triggering day under long or antagonist protocol Exclusion criteria: contraindication to oocyte puncture, oocyte donor, couples treated in viral programs, not speaking French, unable to provide informed consent, a lack of follicles on the day of triggering or patients who had oocyte retrieval at the weekend.	Study group (n=127): The first follicle was aspirated using a 35 cm double-lumen 17-gauge needle and the fluid collected in a tube, after which the tube was changed to collect the flush out from this same follicle using a flushing medium once for each follicle. Control group (n=125) Puncture with 35 cm single-lumen 17-gauge needle. The follicular fluid were collected in tubes without differentiating between the follicles	Primary outcome: No oocyte retrieved No MII oocytes Secondary outcomes: fertilization rate No embryos transferred Clinical pregnancy rate LBR Miscarriage rate	NCT 01329302
De Souza 2021 [14]	Single Brazil	208 randomized and analyzed	Inclusion criteria: Age 34 -42 years Poor responders Exclusion criteria:	Study group (n= 105): 17-gauge double-lumen needles were used, half buffered medium was injected into each follicle, followed by a new aspiration, and the liquid up to 3 times Control group (n=103): single-gauge 19-gauge needles	No of aspirated oocytes No of MII oocytes Capture failure rate Oocyte/ follicle ratio	Not registered
Haines 1989 [15]	Single Australia	36 randomized and analyzed	Unclear inclusion and exclusion	Study group (n=18): A double lumen 17 G; 25 cm needle is used. If the oocyte was not recovered on the first aspirate, the follicle was then flushed and aspirated up to five times Control group (n=18) A single-lumen 17 G, 23.5 cm needle was allocated, each follicle was punctured and aspirated	Number of follicles	Not registered
Haydardedeoglu 2011 [16]	Single Turkey	274 randomized and analyzed	Inclusion criteria: Women with normal ovarian response undergoing IVF Exclusion criteria: Poor responders Women under microdose flare protocol High responders as PCOS	Study group ( n=149): A 17-gauge needle was used to aspirate the follicles then 2 mL flush medium was reinjected and re-aspirated once for each punctured follicle Control group ( n=125): ), a 17-gauge needle was used to aspirate the follicles	No oocyte retrieved No MII oocytes Duration of oocyte retrieval Fertilization rate No of embryos transferred CPR ChPR LBR Implantation rate Cancellation rate hospitalization	NCT 00995280
Haydardedeoglu 2011 [17]	Single Turkey	80 randomized and analyzed	Inclusion criteria: Age 20-43 years Poor responders Exclusion criteria: Natural IVF cycle Monofollicular response Endometrioma	Study group (n=40): A 17-gauge needle was used to aspirate follicles then 2 mL of warmed (37°C) culture medium was injected into each follicle and re-aspirated and re-injected three times for each punctured follicle Control group (n=40): a 17- gauge needle was used to aspirate follicles	Primary outcome: No MII oocytes Secondary outcomes No retrieved oocytes Fertilization rate Implantation rate Duration of procedure CPR LBR	NCT 02391155
Kara 2012 [18]	Single Turkey	200 randomized and analyzed	Inclusion criteria: women undergoing IVF using long protocol	Study group ( n=100): a double-lumen transvaginal oocyte retrieval needle was used to aspirate the follicles then each aspirated follicle was washed with 2 mL flush medium and re-aspirated Control group ( n=100): a single-lumen transvaginal oocyte retrieval needle was used with a single follicle puncture	No retrieved oocytes No MII oocytes Time of procedure Fertilization rate Cancellation rate CPR OPR	No registration
Kingsland 1991 [19]	Single UK	34 randomized and analyzed	Inclusion criteria: Age ≤ 35 years Tubal infertility Exclusion criteria:	Study group (n=18): a JP6L double channeled needle was used for follicle aspiration and then flushing with 10 ml of Earle's balanced salt solution Control group (n=16): a JP6L double channeled needle was used for follicle aspiration	No oocyte retrieved Duration of procedure Fertilization rate CPR OPR	No registration
Lainas 2023 [20]	Single Greece	210 randomized and analyzed	Inclusion criteria Age <43 years BMI 18-35 kg/m2 Women underwent ovarian stimulation for ICSI Exclusion criteria: Single ovary Ovarian pathology Use of IVF for fertilization	Study group (n=105): A 16G double lumen was used to aspirate the follicles Control group (n=105): A 16G double lumen was used to aspirate the follicles if a COC was not retrieved in the initial aspirate, follicular flushing was performed until a COC was retrieved, up to a maximum of five times	Primary outcome: No of COCs Secondary outcomes: Oocyte recovery rate , oocyte maturation rate, fertilization rate , good quality embryos on Day 2 rate procedure time	NCT05473455
Levens 2009 [21]	Single USA	30 randomized and analyzed	Inclusion criteria: Low ovarian response Exclusion criteria: Patients’ ineligible for transvaginal oocyte retrieval secondary to hyporesponse	Study group (n=15): a 35 cm 16-gauge double-lumen transvaginal oocyte retrieval needle was used to aspirate follicle then flushed once with 2 mL of sterile phosphate buffered saline Control group (n=15): a 35 cm 16-gauge single-lumen transvaginal oocyte retrieval needle was used for follicle aspiration	Primary outcome: No oocytes retrieved Secondary outcomes: recovery rate, total number of mature oocytes, maturity rate, fertilization rate, number of embryos transferred, implantation rate, on-going pregnancy rate, and retrieval time	No registration
Malhotra 2020 [22]	Single India	71 randomized and analyzed	Inclusion criteria: Age 22-38 years having 3–5 follicles ≥14 mm on the day of triggering normal uterine cavities Exclusion criteria: endometrioma	Study group (n= 35): A double lumen needle of 17-gauge was used to aspirate the follicles followed by 2 mL of flush with culture medium if no oocyte was retrieved at the direct aspiration up to 3 times Control group (n=36): a single lumen needle of 17 gauge with a suction pressure of 100–110 mm Hg was used to aspirate the follicles	Primary outcome: No oocytes retrieved Secondary outcomes: Time of anesthesia and procedure, fertilization rate, cleavage rate, No of embryos, grade 1 embryos and embryos transferred, failed oocyte recovery, implantation rate, miscarriage rate. CPR LBR	CTRI/2017/07/009062
Méndez Lozano 2007 french	Sigle France	123 randomized and analyzed				
Mok-Lin 2013 [23]	Single USA	50 randomized and analyzed	Inclusion criteria: Poor responders Exclusion criteria: Patients without a planned, fresh embryo transfer, patients undergoing natural IVF and patients with canceled cycles Women randomized in a previous cycle	Study group (n=25): each aspirated follicle was flushed up to four times using a manually pressed syringe with 5 ml of culture media warmed to 378C and re-aspirated using a 16-gauge double-lumen needle Control group ( n=25): a 16-gauge single-lumen oocyte retrieval needle was used to aspirate the follicles with transvaginal ultrasound guidance	Primary outcome: No retrieved oocytes Secondary outcomes: Time of anesthesia and procedure, No of mature oocytes, No of embryos transferred implantation rate CPR LBR	NCT 01558141
Ronchetti 2023 [24]	Single Italy	200 randomized and analyzed	Inclusion criteria: Age 18 -42 years Candidate for IVF or ICSI Exclusion criteria: frozen pelvis from PID or endometrioma	Study group (n=100): a 17-gauge double-lumen needle was used to aspirate and flush the follicles twice Control group (n=100): a 17-gauge Cook® Single Lumen Ovum Aspiration Needle was used to aspirate follicles	Primary outcome: No oocytes retrieved Secondary outcome: No punctured follicles Retrieved / triggered follicle ratio Retrieval time No MII oocytes No embryos transferred Complications CPR OPR	NCT03611907
Salman 2015 [25]	Single Egypt	185 randomized and analyzed	Inclusion criteria: infertile women who underwent IVF/ICSI Exclusion criteria: unclear	Study group (n=92): a 16-gauge double-way tap with (2 ml) injection of Earl's medium till oocyte retrieved or maximum two times Control group (n=93): a 16-gauge single lumen needle used, with suction continue until a small amount of blood-stained fluid appeared in the tubing or flow stop	No retrieved oocytes Operative time No embryos transferred CPR ChPR	No registration
Schwartz 2020 [26]	Single Switzerland	164 randomized and analyzed	Inclusion criteria: Age 18-42 years Candidate for Gn free monofollicular IVF Regular menstrual cycles Exclusion criteria: women with >2 previous embryo transfers without pregnancy, an LH surge on the trigger day, or previous enrolment in the current study	Study group (n=83): a 19-gauge single-lumen needles was used to aspirate the follicle then flush the follicles 5 times with a flushing medium containing heparin Control group (n= 81): a 19-gauge single-lumen needle was used to aspirate the follicle	Primary outcome No MII oocytes Secondary outcomes: No oocytes retrieved No flushes Fertilization rate Transfer rate Implantation rate CPR LBR	NCT 02641808
Scott 1989 [27]	Single USA	44 randomized and analyzed	Inclusion criteria: women underwent IVF Exclusion criteria: unclear	Study group (n=22): aspirating the contents of the follicle and then injecting through the second port enough heparinized Delbecco's solution (1-3 ml) to reexpand the follicle. This volume was then aspirated back into the syringe. This lavage was performed one or more times until the oocyte was recovered or until the follicle Control group ( n= 22): aspirate the follicle with a hand-held 20-ml syringe, remove the needle from the patient, and then aspirate an additional 2 ml of heparinized Delbecco's solution through the system to wash the fluid In both groups the needle has an outer diameter of 1.5 mm and an inner diameter of 1.0 ram. With 4 shallow grooves. The DLN incorporates a needle-within-a-needle design	No of oocytes aspirated No oocyte recovery	No registration
Tan 1992 [28]	Single UK	100	Inclusion criteria: women underwent IVF Exclusion criteria: women developed >25 or <4 follicles wider than 14 mm diameter on the day of triggering	Study group (n=50): follicle aspiration using the JP6L double-channel needle, then flushing with 1.5 mL of media Control group (n= 50): follicle aspiration using the JP6L double-channel needle, the inner channel of the needle was removed so that the needle was converted into a single-channel needle.	No aspirated follicles No oocytes retrieved Time od procedure Fertilization rate Oocyte recovery rate O embryos transferred CPR	No registration
Von horn 2017 [29]	Single Germany	80	Inclusion criteria: Age 18-45 years BMI 18-35 kg/m2 Had ≤ 5 follicles > 10 mm at the end of follicular phase in both ovaries Candidate for IVF/ICSI Exclusion criteria: Women with 1 ovary Difficulty puncturing one or both ovaries	Study group (n=40): Follicles in both ovaries were to be aspirated by a17GSteiner-TanNeedle®withasuctionpressureof180mmHgand then flushed 3 times Control group (n=40): all visible follicles in both ovaries were to be aspirated by the17 G Gynetics ® single-lumen needle with a suction pressure of 180 mmHg	Primary outcome: No of COC Secondary outcomes: Oocyte retrieval rate No MII oocytes No fertilized oocytes Procedure time OPR Pain score	NCT02365350

**Fig 2:**
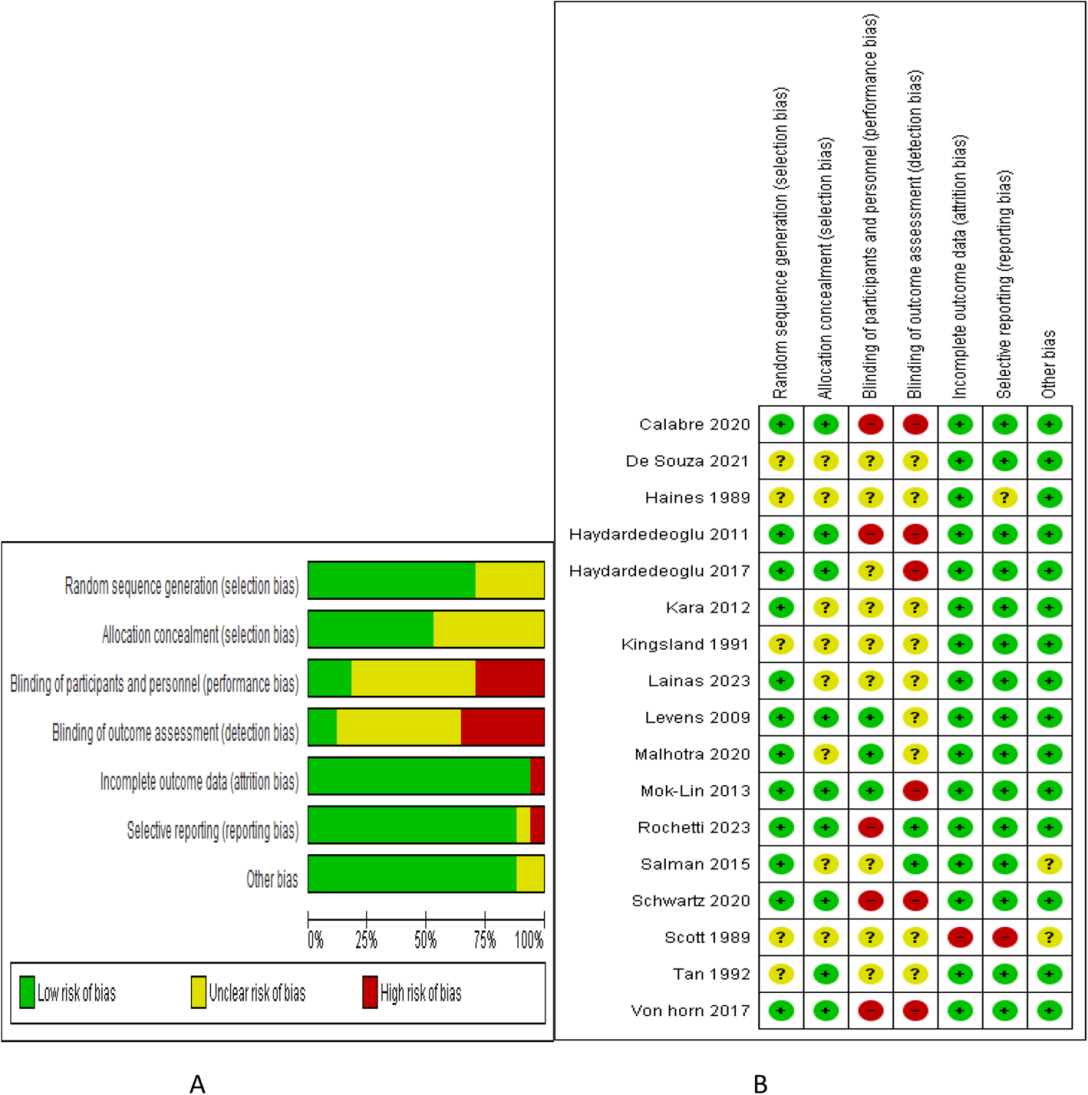
Risk of bias A graph B summary

The risk of bias is described in Figure 2.

### Synthesis of results

**Fig 3:**
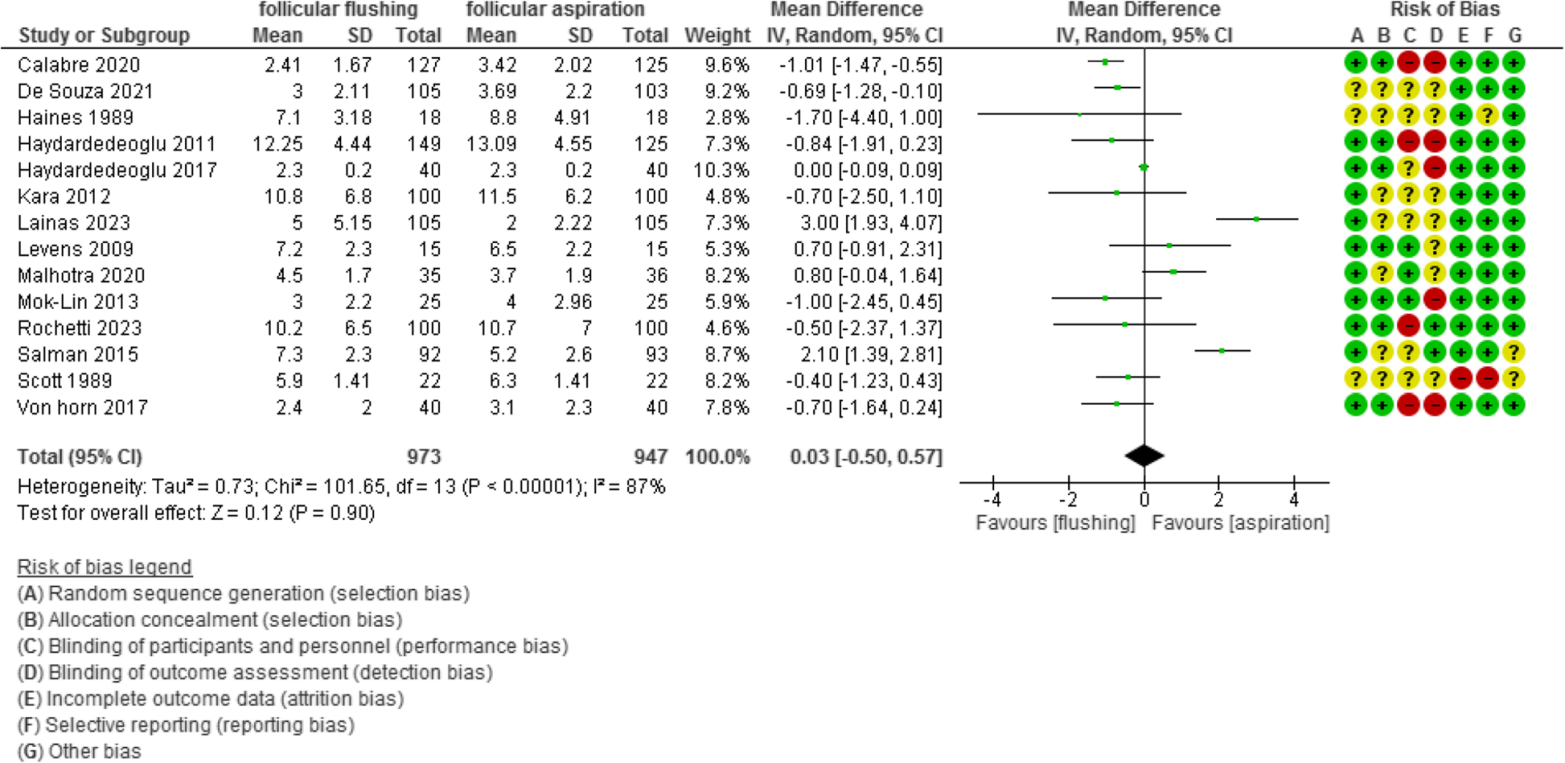
The number of retrieved oocytes

The number of retrieved oocytes was evaluated in 14 studies with 1920 participants (973 were subjected to follicular flushing and 947 were subjected to follicular aspiration) and revealed a mean difference (MD) of 0.03 with [-0.50, 0.57] 95% CI (*P* value =0.9, I^2^ = 87%) (Figure 3).

**Fig 4:**
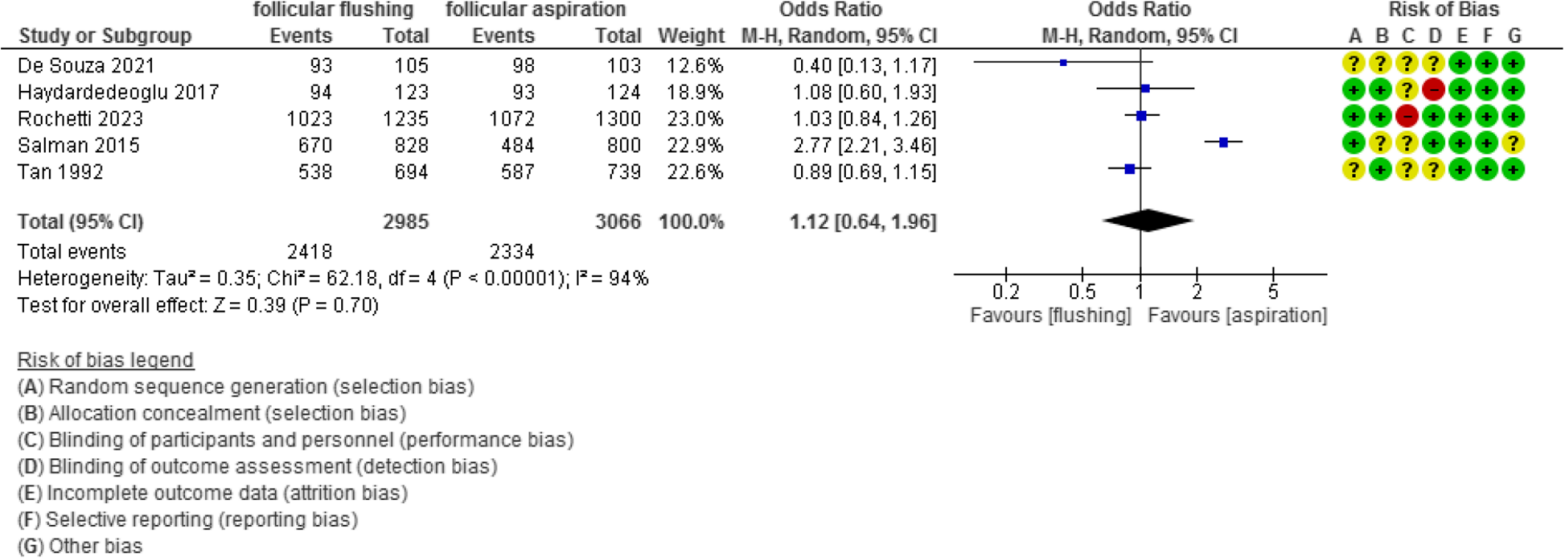
The oocyte/ follicle ratio

The oocyte/ follicle ratio was evaluated in 5 studies with 6051 participants (2985 were subjected to follicular flushing and 3066 were subjected to follicular aspiration) and revealed an Odd Ratio (OR) of 1.12 with [0.64, 1.96]95% CI (*P* value =0.7, I^2^ = 94%) (Figure 4).

**Fig 5:**
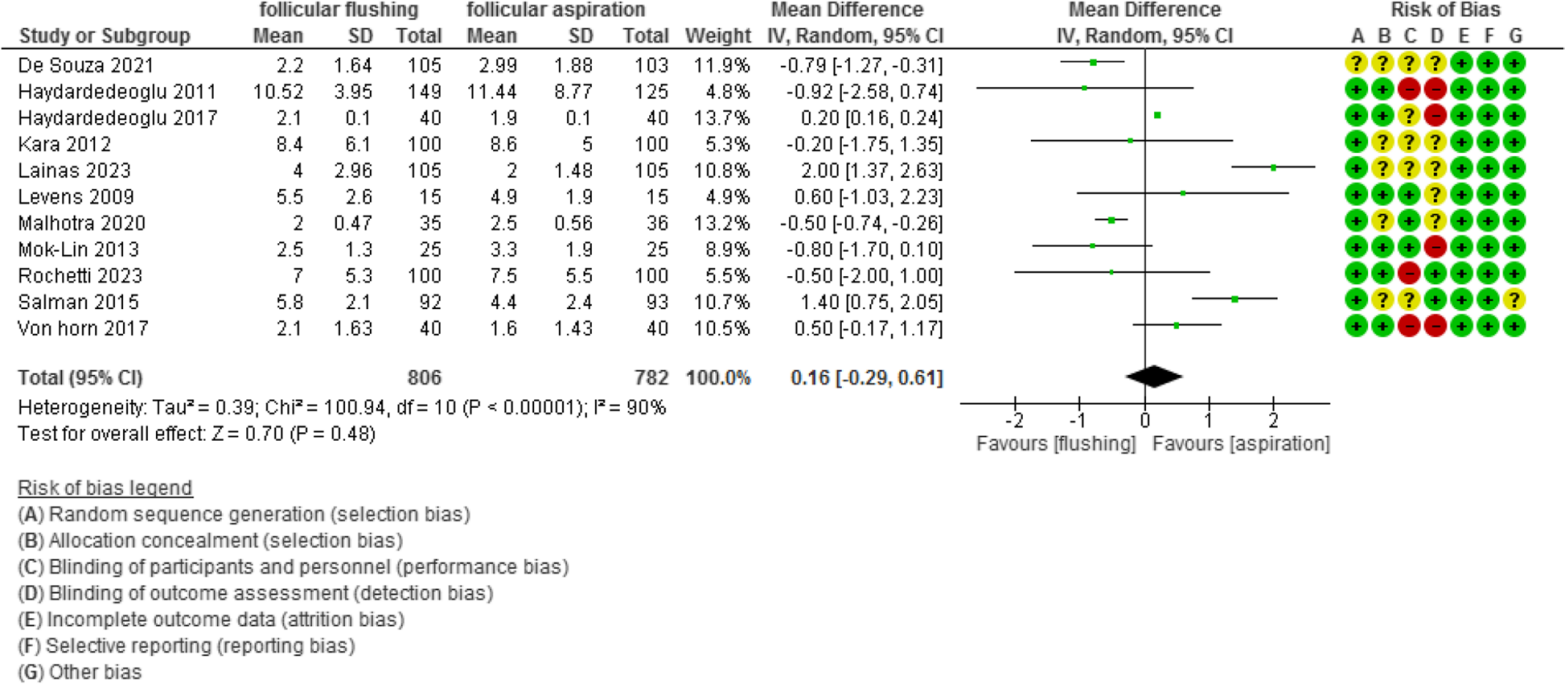
The number of MII oocytes

The number of MII oocytes was evaluated in 11 studies with 1588 participants (806 were subjected to follicular flushing and 782 were subjected to follicular aspiration) and revealed a mean difference (MD) of 0.16 with [-0.29, 0.61] 95% CI (*P* value =0.48, I^2^ = 90%) (Figure 5).

**Fig 6:**
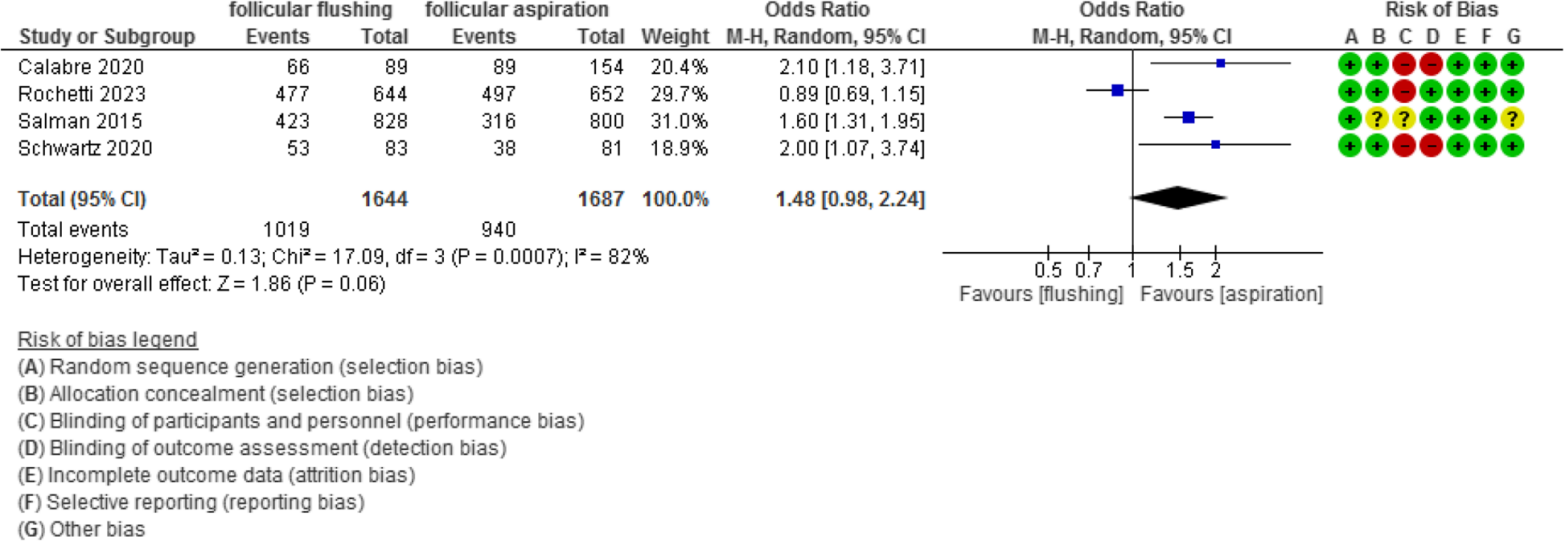
Fertilization rate

The fertilization rate was evaluated in 4 studies with 3331 participants (1644 were subjected to follicular flushing and 1687 were subjected to follicular aspiration) and revealed an Odd Ratio (OR) of 1.48 with [0.98, 2.24] 95% CI (*P* value =0.06, I^2^ = 82%) (Figure 6).

**Fig 7:**
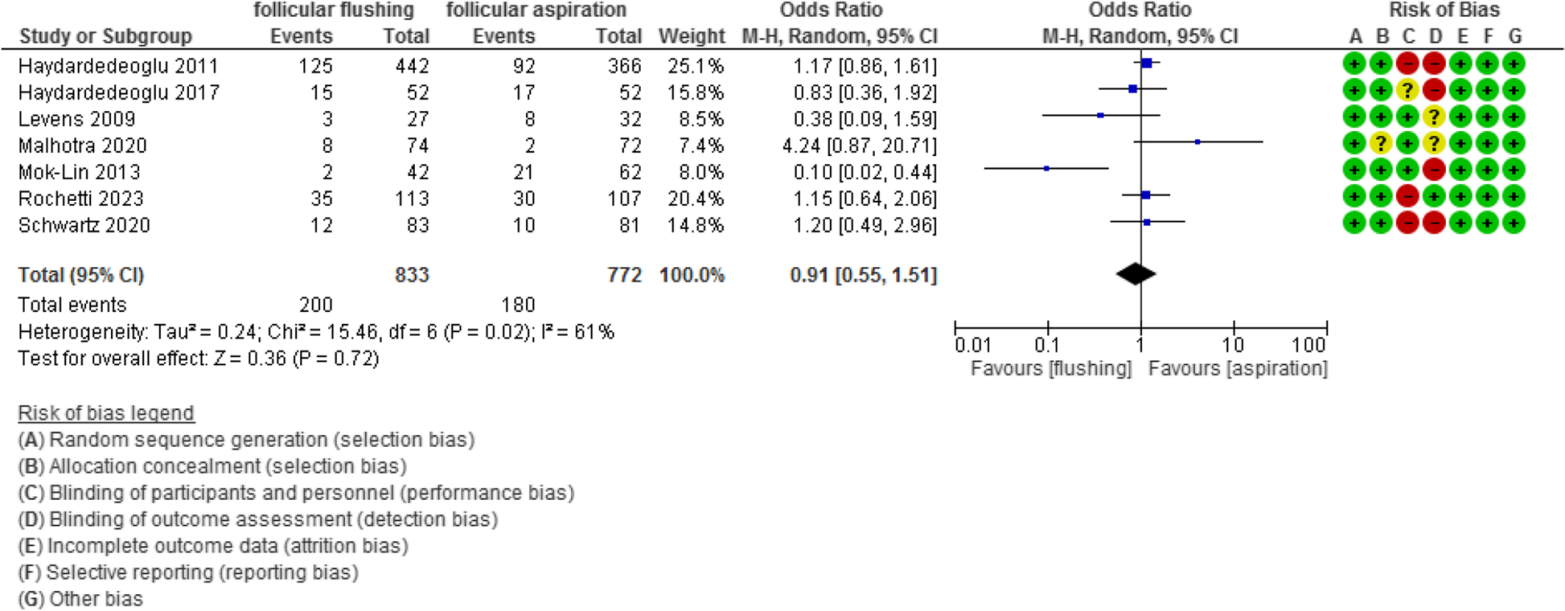
Implantation rate

The implantation rate was evaluated in 7 studies with 1605 participants (833 were subjected to follicular flushing and 772 were subjected to follicular aspiration) and revealed an Odd Ratio (OR) of 0.91 with [0.55, 1.51] 95% CI (*P* value =0.72, I^2^ = 61%) (Figure 7).

**Fig 8:**
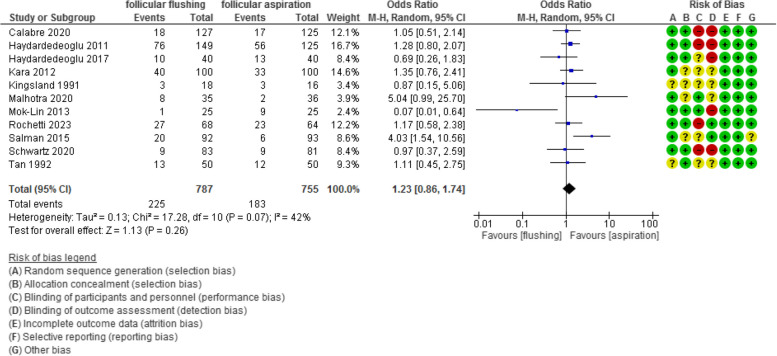
Clinical pregnancy rate

The clinical pregnancy rate was evaluated in 11 studies with 1542 participants (787 were subjected to follicular flushing and 755 were subjected to follicular aspiration) and revealed an Odd Ratio (OR) of 1.23 with [0.86, 1.74] 95% CI (*P* value =0.26, I^2^ = 42%) (Figure 8).

**Fig 9:**
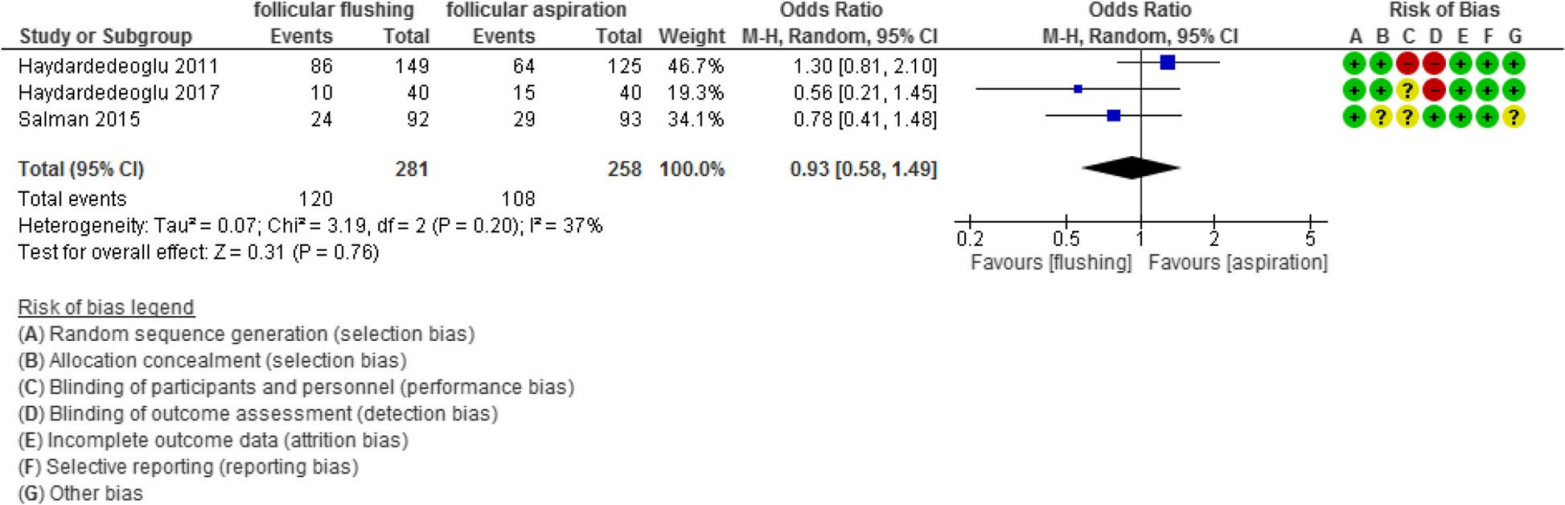
Chemical pregnancy rate

The chemical pregnancy rate was evaluated in 3 studies with 539 participants (281 were subjected to follicular flushing and 258 were subjected to follicular aspiration) and revealed an Odd Ratio (OR) of 0.93 with [0.58, 1.49] 95% CI (*P* value =0.76, I^2^ = 37%) (Figure 9).

**Fig 10:**
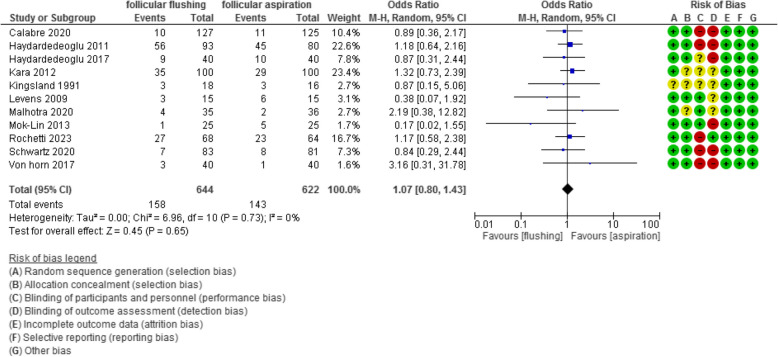
Ongoing pregnancy/live birth rate

The ongoing pregnancy /livebirth rate was evaluated in 11 studies with 1266 participants (644 were subjected to follicular flushing and 622 were subjected to follicular aspiration) and revealed an Odd Ratio (OR) of 1.07 with [0.80, 1.43] 95% CI (*P* value =0.65, I^2^ = 0%) (Figure 10).

**Fig 11:**
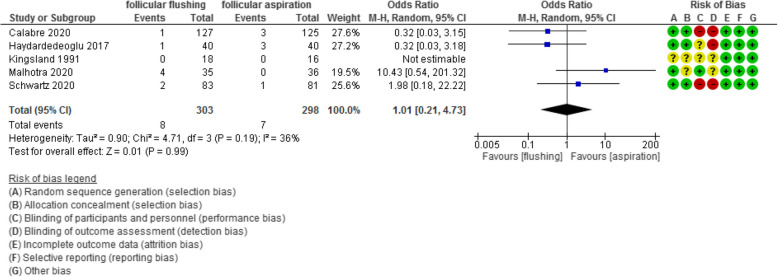
Miscarriage rate

The miscarriage rate was evaluated in 5 studies with 601 participants (303 were subjected to follicular flushing and 298 were subjected to follicular aspiration) and revealed an Odd Ratio (OR) of 1.01 with [0.21, 4.73] 95% CI (*P* value =0.99, I^2^ = 36%) (Figure 11).

**Fig 12:**
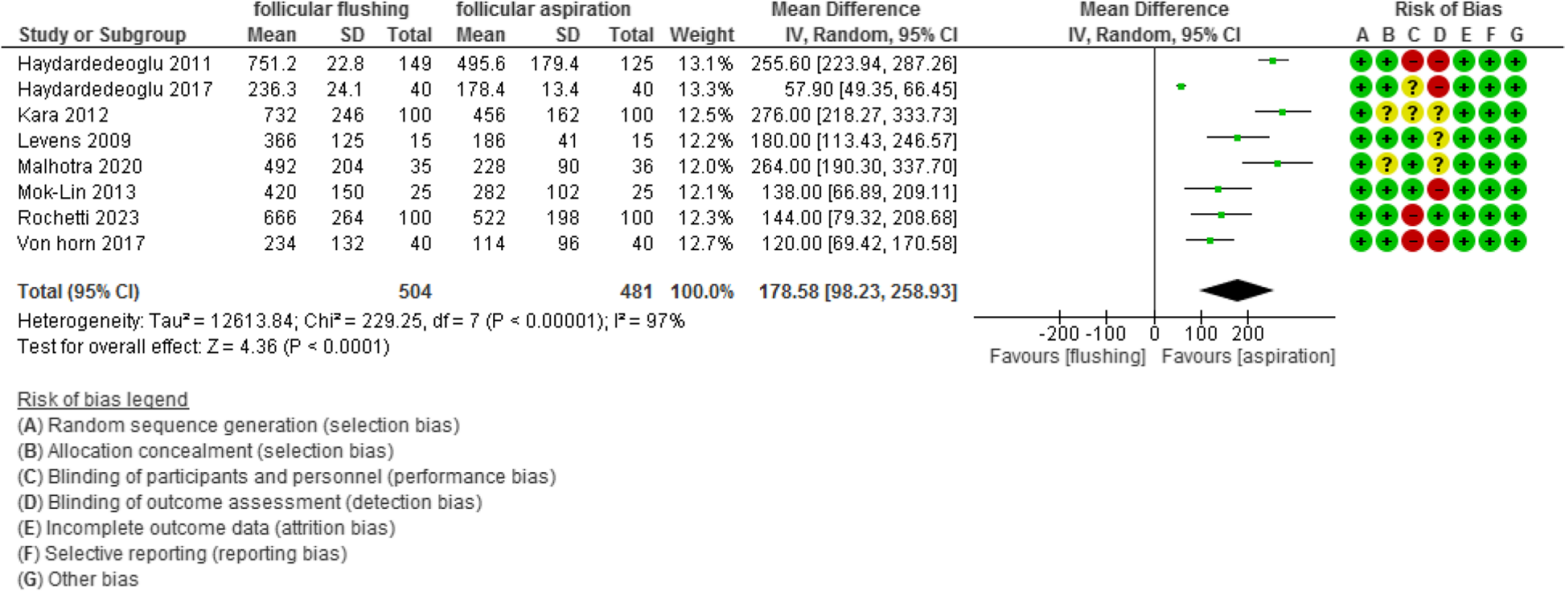
Time of the procedure

The time of the procedure was evaluated in 8 studies with 985 participants (504 were subjected to follicular flushing and 481 were subjected to follicular aspiration) and revealed a mean difference (MD) of 178.58 with [98.23, 258.93] 95% CI (*P* value <0.001, I^2^ = 97%) (Figure 12).

**Fig 13:**
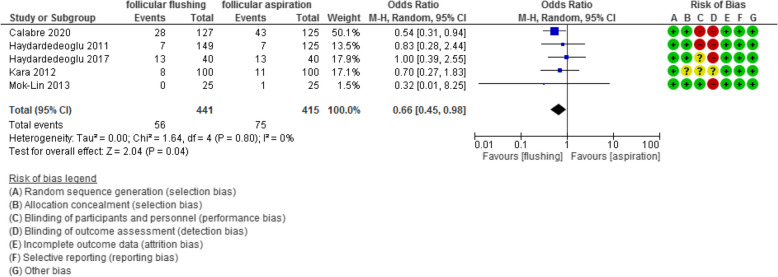
Cancellation rate

The cycle cancellation rate was evaluated in 5 studies with 856 participants (441 were subjected to follicular flushing and 415 were subjected to follicular aspiration) and revealed an Odd Ratio (OR) of 0.66 with [0.45, 0.98] 95% CI (*P* value =0.04, I^2^ = 0%) (Figure 13).

Subgroup analysis for different outcomes according to the ovarian response of participants and number of flushes is described in Table 2 and the quality of evidence using GRADE analysis is described in Table 3.

**Table 2:** Subgroup analysis of outcomes

			No of studies	No of participants	Effect estimates
Number of oocytes retrieved	Participants ovarian response	Poor responders	7	771	-0.32 [-0.83, 0.19]
		Normal responders	1	274	-0.84 [-1.91, 0.23]
		Unspecified	6	875	0.50 [-0.96, 1.95]
	No of flushes	One	5	800	-0.70 [-1.16, -0.24]
		Two	2	385	0.95 [-1.58, 3.48]
		Three	4	439	-0.14 [-0.67, 0.39]
		Four	1	50	-1.00 [-2.45, 0.45]
		Five	2	246	0.82 [-3.77, 5.41]
Oocyte/follicle ratio	Participants ovarian response	Poor responders	2	455	0.73 [0.28, 1.91]
		Unspecified	3	5596	1.37 [0.68, 2.74]
	No of flushes	Two	2	4163	1.68 [0.64, 4.45]
		Three	2	455	0.73 [0.28, 1.91]
		Six	1	1433	0.89 [0.69, 1.15]
Number of MII oocytes	Participants ovarian response	Poor responders	6	519	-0.20 [-0.68, 0.28]
		Normal responders	1	274	-0.92 [-2.58, 0.74]
		Unspecified	4	795	0.90 [-0.12, 1.92]
	No of flushes	One	3	504	-0.16 [-1.09, 0.77]
		Two	2	385	0.57 [-1.27, 2.42]
		Three	4	439	-0.17 [-0.70, 0.37]
		Four	1	50	-0.80 [-1.70, 0.10]
		Five	1	210	2.00 [1.37, 2.63]
Fertilization rate	Participants ovarian response	Poor responders	1	164	2.00 [1.07, 3.74]
		Unspecified	2	2924	1.20 [0.68, 2.13]
	No of flushes	One	1	243	2.10 [1.18, 3.71]
		Two	2	2924	1.20 [0.68, 2.13]
		Five	1	164	2.00 [1.07, 3.74]
Implantation rate	Participants ovarian response	Poor responders	4	413	0.60 [0.16, 2.28]
		Normal responders	1	808	1.17 [0.86, 1.61]
		Unspecified	1	220	1.15 [0.64, 2.06]
	No of flushes	One	2	867	0.83 [0.30, 2.32]
		Two	1	220	1.15 [0.64, 2.06]
		Three	2	250	1.63 [0.34, 7.89]
		Four	1	104	0.10 [0.02, 0.44]
Clinical pregnancy rate	Participants ovarian response	Poor responders	5	617	0.90 [0.40, 2.01]
		Normal responders	1	274	1.28 [0.80, 2.07]
		Unspecified	5	651	1.47 [0.94, 2.28]
	No of flushes	One	3	726	1.25 [0.90, 1.74]
		Two	2	317	2.08 [0.62, 6.95]
		Three	2	151	1.67 [0.24, 11.67]
		Four	1	50	0.07 [0.01, 0.64]
		Five	2	198	0.95 [0.40, 2.23]
		Six	1	100	1.11 [0.45, 2.75]
Ongoing/livebirth rate	Participants ovarian response	Poor responders	7	727	0.85 [0.52, 1.38]
		Normal responders	1	173	1.18 [0.64, 2.16]
		Unspecified	3	366	1.23 [0.79, 1.91]
	No of flushes	One	4	655	1.10 [0.76, 1.60]
		Two	1	132	1.17 [0.58, 2.38]
		Three	3	231	1.26 [0.55, 2.90]
		Four	1	50	0.17 [0.02, 1.55]
		Five	2	198	0.85 [0.34, 2.11]
Time of procedure	Participants ovarian response	Poor responders	5	311	147.67 [73.08, 222.26]
		Normal responders	1	274	255.60 [223.94, 287.26]
		Unspecified	2	400	210.84 [81.49, 340.19]
	No of flushes	One	3	504	241.99 [194.99, 288.98]
		Two	1	200	144.00 [79.32, 208.68]
		Three	3	231	141.59 [38.34, 244.84]
		Four	1	50	138.00 [66.89, 209.11]
Cancellation rate	Participants ovarian response	Poor responders	3	382	0.63 [0.39, 1.00]
		Normal responders	1	274	0.83 [0.28, 2.44]
		Unspecified	1	200	0.70 [0.27, 1.83]
	No of flushes	One	3	726	0.61 [0.39, 0.95]
		Three	1	80	1.00 [0.39, 2.55]
		Four	1	50	0.32 [0.01, 8.25]

**Table 3:** GRADE quality of evidence

Outcome	No studies	Risk of bias	Inconsistency	Indirectness	Imprecision		Publication bias	Quality
					Sample size	Wide CI		
Number of oocytes retrieved	14	N	S	N	1920	N	N	Moderate
Oocyte/follicle ratio	5	N	S	N	6051	N	N	Moderate
Number of MII oocytes	11	N	N	N	1588	N	N	High
Fertilization rate	4	N	S	N	3331	N	N	Moderate
Implantation rate	7	N	S	N	1605	N	N	Moderate
Clinical pregnancy rate	11	N	N	N	1542	N	N	High
Chemical pregnancy rate	3	N	N	N	539	N	N	Moderate
Ongoing/livebirth rate	11	N	N	N	1266	N	N	High
Miscarriage rate	5	N	N	N	601	N	N	Moderate
Time of procedure	8	N	N	N	985	N	N	High
Cancellation rate	5	N	N	N	856	N	N	High

*N* not serious, *S* serious

## Discussion

This meta-analysis confirmed that follicular flushing during oocyte retrieval did not improve any of the IVF cycle outcomes except the reduction of cycle cancellation rate (high evidence). The non improved outcomes included the number of retrieved oocytes (moderate evidence), the oocyte retrieved over the aspirated follicles ration (moderate evidence), the number of MII oocytes (high evidence), the fertilization rate (moderate evidence), implantation rate (moderate evidence), clinical pregnancy (high evidence), chemical pregnancy (moderate evidence), ongoing pregnancy/livebirth (high evidence), and miscarriage rates (moderate evidence).

Our review confirmed high evidence that the procedure of follicular flushing was associated with significant prolongation of the procedure of ovum pick up.

These findings were constant through all subgroup analysis with few exceptions. These include the higher number of oocytes retrieved in the flush group if the flush was done once, the fertilization rate being higher in the flush group in poor responders and in women who underwent one and five flushes, the implantation and clinical pregnancy rates being higher in the flush group after four flushes (however that was derived from Moklin and colleagues study only).

The lower cancellation rate was significantly evident in poor responders and after one flush only while it shows non-significant differences in other women.

### Strengths and limitations

Our meta-analysis provides the largest evidence about the value of follicular flushing during ovum pick up. All available RCTs without any language limitations were included. Careful and complete data extraction, meticulous risk of bias assessment for all individual studies were done by 2 authors independently. All authors for the included articles were contacted via email for clarifications and any missing data. A GRADE assessment of the quality of evidence for all outcomes was achieved. Extensive subgroup data analysis was calculated for all the available outcomes according to the ovarian reserve nature of included participants and the number of flushes.

The main limitations of this significant heterogeneity among the included studies. Most of the studies lack blind nature through their risk of bias assessment. Not all studies reported the same outcomes and most of the studies focused on the number of oocytes and other laboratory data with less concentration on the clinical outcomes of the procedure, especially livebirth rates. We tried to overcome this heterogeneity through analysis of data using the random effect model and through extensive subgroup analysis. Although all authors were contacted several times, only few authors responded for data clarification. In this review, we failed to report the side effects and complications of the procedures as they were rarely reported by the included studies. However, that was not considered as a major limitation as the process of ovum pick up is relatively safe.

### Comparison with existing reviews

The Martini and colleagues systematic review included 11 studies (1,178 cases). They found that follicular flushing was not associated with improvement in either livebirth or clinical pregnancy rates. They reported a lower number of retrieved oocytes and MII oocytes and longer duration of the procedure in women who underwent follicular flushing compared to those who underwent direct aspiration. Compared to our systematic reviews, not all outcomes were reported, and subgroup analysis was not done due to inclusion of smaller number of studies [12].

Neumann and colleagues in 2023 conducted a systematic review to assess the value of follicular flushing in poor responders. It included 6 RCTs. They reached a conclusion that the effect of follicular flushing in poor responders is uncertain. Their review included only 6 studies, and the clinically related outcomes as clinical pregnancy and livebirth rates were not assessed [30].

A recent Cochrane review included 15 studies (1643 women) compared to 17 studies (2218 participants) in our review. The authors concluded that the value of follicular flushing is questionable on laboratory outcomes such as the numbers of retrieved oocytes, total number, and number of cryopreserved embryos and clinical outcomes such as clinical pregnancy, livebirth, and miscarriage rates. Although the authors evaluated most of the clinical outcomes, other outcomes such as fertilization, implantation and cycle cancellation rates were not evaluated. Also, extensive subgroup analysis was not done [31].

## Conclusion

This systematic review concluded that the practice of follicular flushing was not associated with improvement of IVF outcomes named the number of oocytes retrieved, the oocyte / follicle ratio, fertilization, implantation, clinical pregnancy, chemical pregnancy, live birth, and miscarriage rates. The cycle cancellation rate showed a significant improvement in follicular flushing in women with POR. The follicular flushing was associated with prolongation of the time of ovum pick up with expected prolongation of the anesthesia time and subsequently its complications and increase in the costs.

According to the current evidence, follicular flushing is not recommended during ovum pick up. We recommend a well-organized multicenter blinded RCTs conduction with standardization of the suction pressure and the number of flushes for each follicle to reach a solid conclusion about the use of follicular flushing especially in women with considerable risk of unfavorable outcomes as poor responders.

## Data Availability

No datasets were generated or analysed during the current study.
